# Profiles of criteria and non-criteria anti-phospholipid autoantibodies are associated with clinical phenotypes of the antiphospholipid syndrome

**DOI:** 10.1186/s13317-020-00131-3

**Published:** 2020-05-15

**Authors:** Ilan Volkov, Luciana Seguro, Elaine P. Leon, László Kovács, Dirk Roggenbuck, Peter Schierack, Boris Gilburd, Andrea Doria, Maria G. Tektonidou, Nancy Agmon-Levin

**Affiliations:** 1 Clinical Immunology, Angioedema and Allergy Unit, Zabludowicz Center for Autoimmune Diseases, The Chaim Sheba Medical Center, Tel-Hashomer, 52621 Israel; 2 Zabludowicz Center for Autoimmune Diseases, Sheba Medical Center, Tel-Hashomer, 52621 Israel; 3 Rheumatology Division, Hospital Das Clinicas HCFMUSP, Faculdade de Medicina, Universidade de Sao Paulo, Sao Paulo, SP Brazil; 4 Department of Rheumatology and Immunology, Faculty of Medicine, University of Szeged, Szeged, Hungary; 5 Institute of Biotechnology, Faculty Environment and Natural Sciences, Brandenburg University of Technology Cottbus-Senftenberg, Cottbus, Germany; 6 Rheumatology Unit, Department of Medicine, University of Padova, Padova, Italy; 7 Rheumatology Unit, First Department of Propaedeutic Internal Medicine, School of Medicine, National and Kapodistrian University of Athens, Athens, Greece; 8 Sackler Faculty of Medicine, Tel-Aviv University, Tel Aviv, Israel

**Keywords:** Anti-phospholipid syndrome, Antiphospholipid antibody, Anti-cardiolipin, Anti-β2GP1, Anti-prothrombin, Phosphatidic acid, Anti-phosphatidylcholine, Anti-phosphatidylethanolamine, Anti-phosphatidylglycerol, Anti-phosphatidylinositol, Anti-phosphatidylserine, Anti-annexin 5, Phenotypes

## Abstract

**Background:**

Specific anti-phospholipids antibodies (aPLs) are used as classification criteria of the antiphospholipid syndrome (APS). These aPLs, although essential for diagnosis, do not predict disease phenotypes, which may require specific therapies. Non-criteria aPLs are rarely evaluated and their role is yet to be defined. In the current study, we aimed to examine the association between criteria and non-criteria aPLs and APS phenotypes.

**Methods:**

Serum samples from 188 subjects, 130 APS patients and 58 controls were analyzed for the presence of 20 aPLs (IgG and IgM isotypes to cardiolipin (CL), beta2-glycoprotein1 (β2GP1), phosphatidic acid (P-acid), phosphatidylcholine (PC), phosphatidylethanolamine (PE), phosphatidylglycerol (PG), phosphatidylinositol (PI), phosphatidylserine (PS), annexin-5 (AN) and prothrombin (PT) using a line immunoassay (GA Generic Assays, Germany). Sero-positivity to the different aPLs/aPLs profiles was correlated to APS phenotypes (i.e. arterial thrombosis, CNS manifestations, venous thrombosis, relapsing disease, obstetric morbidity).

**Results:**

In this cohort, arterial thrombosis was associated with accumulative number of ≥ 7/20 aPLs evaluated (OR 4.1; CI 95% 1.9–96, p = 0.001) as well as the sole presence of aPT (IgG) (OR 2.3;CI 95% 1.1–5.1, p = 0.03). CNS manifestations were linked with a profile of 4 aPLs (IgG): aPT, aPG, aPI and aAN (OR 2.6;CI 95% 1.1–6.3, p = 0.03). Symptom-free period of ≥ 3 years was linked with lower number of aPLs and the presence of aPI (IgG) (OR 3.0;CI 95% 1.08–8.1, p < 0.05) or aAN (IgG) (OR 3.4;CI 95% 1.08–10.9, p < 0.05). APS related pregnancy morbidity correlated with a profile of 2 aPLs (IgG): aCL and aPS (OR 2.9; CI 95% 1.3–6.5, p < 0.05) or the sole presence of aAN (IgG) (OR 2.8; CI 95% 1.02–8, p = 0.05).

**Conclusion:**

In this study, we observed an association between specific criteria/non-criteria aPLs or aPLs profiles and clinical phenotypes of APS. Our data suggest that examination of a wider variety of aPLs may allow better characterization of APS.

## Background

The antiphospholipid syndrome (APS) is an acquired autoimmune disorder characterized by thrombotic events, obstetric morbidity and a myriad of systemic manifestations induced by the persistent presence of autoantibodies directed at phospholipids or phospholipid-binding proteins (aPLs) [1–4] APS classification criteria, defined in 2006, are currently in use, although their role for diagnosis or assessment of specific APS-related manifestations is less clear [6, 7]. Nevertheless, in the last decade, the significance of aPL persistency and accumulation (i.e. the co-presence of criteria aPLs: anti-cardiolipin (aCL), anti-beta2-glycoprotein1 (aβ2GP1) of the IgG or IgM subtypes and circulating lupus anticoagulant (LAC)) was established, particularly regarding the risk of APS evolvement. Moreover, sero-positivity of all three-classification criteria-aPLs termed the “triple positive”-variant is linked with a more aggressive disease [8–10]. The latter may require specific therapeutic interventions such as enhanced anti-coagulations or addition of other treatment modalities [8, 11–15]. In contrast transient appearance of aPLs during acute thrombosis or infections may not require intervention.

aPLs encompass also a spectrum of non-criteria aPLs which are typically not evaluated [4, 16]. Currently more than 30 different aPLs have been defined some of which bind directly to negatively charged phospholipids (e.g. phosphatidylinositol, phosphatidylserine) while others react with phospholipid binding proteins (e.g. distinct domains of β2GP1, prothrombin, annexin-V) [17–19]. The pathogenic role of non-criteria aPLs as well as their importance in defining APS phenotypes is yet to be revealed. Nevertheless, such roles were described for some non-criteria aPLs, for instance anti-phosphatidylethanolamine (aPE) and anti-phosphatidylserine (aPS) with recurrent pregnancy losses [17–21] or anti-phosphatidylserine/prothrombin (aPS/PT) with thrombosis [21]. Notably, inconsistencies regarding criteria and non-criteria aPLs have been reported [16] and most studies evaluated a single or a few non-criteria aPLs, frequently using different diagnostic platforms, which may be difficult to compare. Lately, a new technique for aPLs testing was developed, using a line immunoassay (LIA), a multiplex method that permits estimation of a relatively large profile of aPLs concomitantly [6, 22]. This novel assay technique appears to discriminate aPLs associated with APS from aPLs detected during infectious diseases and even asymptomatic carriers and may detect specific binding of aβ2GP1 to domain1 (D1) of the β2GP1 [16, 23–25].

Hence, in the current study we evaluated the presence of 20 criteria and non-criteria aPLs amid a cohort of well-defined APS patients and the relationships between aPLs sero-positivity and clinical phenotypes of disease.

## Patients and methods

### Patients

In this case–control multicenter study, we evaluated serum samples from 130 APS patients and 58 geographically matched controls including 40 healthy individuals and 18 patients diagnosed with sepsis that may induce aPLs positivity transiently. Serum samples were stored at − 70 °C prior to their analysis. Diagnosis of APS was defined by the treating specialists, according to the APS classification criteria [5]. Data regarding prior aPL serology (e.g. lupus anti coagulants detected according to international guidelines, and anti-cardiolipin and anti B2GPI antibodies detected by different methods than line blot), APS clinical manifestations/phenotypes as arterial thrombosis, CNS manifestations, venous thrombosis, latency period from last thrombotic events, pregnancy morbidity as well as age, gender and other concomitant autoimmune diseases (i.e. primary or secondary APS) were collected from medical files prior to inclusion in this study and analyzed respectively. The study received approval by the ethics committee (Sheba medical center nu. 4784) and fulfilled the ethical guidelines of the declaration of Helsinki (Edinburgh 2000).

### Methods

We analyzed all sera samples for the presence of 20 different aPLs of the IgG and IgM isotypes directed at 10 antigens namely: cardiolipin (CL), beta-2 glycoprotein1 (β2GP1), phosphatidic acid (P-acid), phosphatidylcholine (PC), phosphatidylethanolamine (PE), phosphatidylglycerol (PG), phosphatidylinositol (PI), phosphatidylserine (PS), annexin 5 (AN) and prothrombin (PT) using a LIA (GA Generic Assays, Germany), as previously described [22, 25]. Briefly, the LIA strip is a hydrophobic membrane (polyvinylidenedifluoride, serving as a solid phase) that contains 11 lines: 10 with different APS-related autoantigenic targets and one for the positive control. Each strip was manually positioned in diluted sera according to the manufacturer’s instructions. Following aPL binding to the target antigen on the solid-phase membrane, it underwent 30 min of washing in room temperature. In the second step, anti-human antibodies, conjugated with horseradish peroxidase were added for 15 min of incubation followed by an additional washing step. Once the horseradish peroxidase converted the colorless substrate into purple, the strips were densitometrically analyzed utilizing a scanner and data interpreted by a software supplied by the manufacturer (GA Generic Assays, Germany). The latter provides results on a scale of 0 to (+ 3) and consider positive reactivity for ≥ (+ 1) defined by the 99%. As this method was recently developed, and differences between populations have been reported, we assessed positivity in our cohort also according to the analysis of healthy subjects in our population. Hence we considered positive only titers levels detected in less or equal to 5% of our healthy control group. Hence, 7 aPLs namely: aPG IgG, aPI IgG, aCL IgM, aPE IgM, aPI IgM, aPG IgM and aβ2GP1 IgM were considered positive if they were higher or equal to (+ 1) while the others were considered positive if the levels were higher or equal to (+ 2). LAC positivity was determined as recorded in the medical files, and was defined prior to initiation of treatment with warfarin.

### Statistical methods

The collected data was transferred and processed using Microsoft excel version 2007 (Microsoft Corp, Seattle WA) and JMP version 7.0 (SAS institute, Cary, NC, USA). The statistical program SPSS 13.0 (SPSS, Chicago, IL, USA) was used for statistical analysis. For comparison between groups as well as correlations with clinical manifestations, we used the Fisher’s exact test, student’s T-test and Pearson Chi Square as appropriate. Values of p less than 0.05 were regarded statistically significant.

## Results

We studied 130 APS patients and 58 controls, of which 40 were healthy subjects and 18 disease controls diagnosed with sepsis. In our cohort of APS, patients’ prior serology was noticeable for LAC detected in 84% and “triple positivity” (i.e. sero-positivity for aCL and a β2GP1 and LAC) in 55% of patients (Table 1). Clinical and serological data obtained from medical files prior to inclusion in this study were collected and analyzed, respectively (Table 1).

**Table 1:** Description of study cohort

Clinical and serological manifestations	APS patients (n = 130)	All controls (n = 58)	Healthy controls N = 40	Sepsis control n = 18
Age (years ± SD)	43.8 ± 13.2	37.7 ± 10.7	37 ± 11.7	39 ± 9
Gender (Female)	79.2%	66%	80%	30%
Time from diagnosis (years ± SD)	8.3 ± 7.4	NA		
Secondary APS	51.5%	NA		
Arterial thrombosis	47.7%	NA		
CNS manifestations	35.4%	NA		
Venous thrombosis	59.2%	NA		
Pregnancy morbidity	29.2%	NA		
(LAC) positivity	84.0%	NA		
Triple positive (aCL + aβ2GP1 + LAC)	55.0%	NA		

Data retrieved from medical files

*APS* antiphospholipid antibody syndrome, *CNS* central nervous system, *NA* not applicable, *aCL* antibody to cardiolipin, *aβ2GP1* antibody to beta2-glycoprotein1, *LAC* lupus anticoagulant

### Prevalence of aPLs among APS patients and controls

In this study, the sensitivity of the LIA including all aPLs of both IgM and IgG isotypes was 83% (49% for the IgM, and 69% for the IgG isotype). Regarding non-criteria aPLs, the specificity of the test was 100% for the IgM isotype and 95% for the IgG antibodies compared to healthy controls. aPLs were more prevalent among APS patients compared to our entire control groups as well as the healthy and diseased control subjects with sepsis separately (Table 2). Among controls, some aPLs were numerically more prevalent in the subgroup of patients suffering from sepsis compared to healthy controls, whereas a statistical significance was reached only for aβ2GP1 IgG detected in 1/40 (2.5%) healthy controls *vs.* 4/18 (22.2%) septic patients (p = 0.03).

**Table 2:** Prevalence of different anti-phospholipid antibodies (aPLs) among patients with the antiphospholipid syndrome (APS) and controls

APLs		APS patients N = 130	All controls N = 58	p value APS vs All controls	Healthy controls (HC) N = 40	p value APS vs HC	Sepsis patients (SP) N = 18	p value APS vs SP
CL	IgM	*34 (26%)*	*1 (2%)*	*0.001*	*0*	*< 0.001*	*1 (5%)*	*0.07*
	IgG	*77 (59%)*	*1 (2%)*	*0.001*	*0*	*< 0.001*	*1 (5%)*	*< 0.001*
P-acid	IgM	*17 (13%)*	*0*	*0.001*	*0*	*< 0.05*	0	NS
	IgG	*73 (56%)*	*0*	*0.001*	*0*	*< 0.001*	*0*	*< 0.001*
PE	IgM	4 (3%)	0	NS	0	NS	0	NS
	IgG	3 (2%)	0	NS	0	NS	0	NS
PG	IgM	4 (3%)	0	NS	0	NS	0	NS
	IgG	*50 (38%)*	*0*	*0.001*	*0*	*< 0.001*	*0*	*< 0.001*
PI	IgM	*11 (8%)*	*0*	*0.01*	*0*	*0.06*	0	NS
	IgG	*59 (45%)*	*1 (2%)*	*0.001*	*1 (2%)*	*< 0.001*	*0*	*< 0.001*
PS	IgM	*16 (12%)*	*0*	*0.003*	*0*	*< 0.05*	0	NS
	IgG	*73 (56%)*	*0*	*0.001*	*0*	*< 0.001*	*0*	*< 0.001*
AN	IgM	2 (1%)	1 (2%)	NS	0	NS	1 (5%)	NS
	IgG	*36 (27%)*	*5 (9%)*	*0.003*	*2 (5%)*	*< 0.01*	*3 (16%)*	*NS*
β2GP1	IgM	*42 (32%)*	*6 (10%)*	*0.003*	*4 (10%)*	*< 0.001*	*2 (10%)*	*NS*
	IgG	*86 (66%)*	*5 (9%)*	*0.001*	*1 (2%)*	*< 0.001*	*4 (22%)*	*< 0.001*
PT	IgM	2 (1%)	0	NS	0	NS	0	NS
	IgG	*40 (30%)*	*1 (2%)*	*0.001*	*0*	*< 0.001*	*1 (5%)*	*< 0.05*

*HC* healthy controls, *SP* sepsis patients, *CL* cardiolipin, *β2GP1* beta2-glycoprotein1, *P-acid* phosphatidic acid, *PC* phosphatidylcholine, *PE* phosphatidylethanolamine, *PG* phosphatidylglycerol, *PI* phosphatidylinositol, *AN* annexin 5, *PS* phosphatidylserine, *PT* prothrombin, *NS* non-significant

### aPLs antibodies/profiles and phenotypes of APS

We analyzed the interactions of each aPL as well as different combinations of aPLs (profiles) with the following clinical phenotypes of APS: arterial thrombosis, CNS manifestations, venous thrombosis, latency period from last thrombotic event and pregnancy morbidity (e.g. recurrent early abortion, premature delivery etc.) as summarized in Table 3. In addition, we correlated serology with demographics (i.e. age, gender) and the presence of other autoimmune diseases, which in the vast majority of cases was systemic lupus erythematosus (i.e. primary or secondary APS).

**Table 3:** APS phenotypes correlation with aPLs and aPLs profiles

APS phenotypes	Associated aPLs and clinical manifestations	O.R.
Arterial thrombosis	> 7 any aPLs^a^	4.1 [CI 95% 1.9–96]
	aPT IgG	2.3 [CI 95% 1.1–5.1]
	Primary APS	2.2 [CI 95% 1.1–4.5]
CNS manifestations	aPT + aPG + aPI and aAN (IgG)	2.6 [CI 95% 1.1–6.3]
Venous thrombosis	aP-acid IgM	0.3 [CI 95% 0.1–0.9]
Event free period > 3 years from last thrombotic events	aPI IgG	3 [CI 95% 1.08–8.1]
	aAN IgG	3.4 [CI 95%1.08–10.9]
Pregnancy morbidity	aCL IgG and aPS IgG	2.9 [CI 95% 1.3–6.5]
	anti-AN IgG	2.8 [CI 95% 1.02–8]
	Arterial thrombosis	3.3 [CI 95% 1.5–7.2]
	CNS manifestations	3.9 [CI 95% 1.7–9]
	Secondary APS	2.3 [CI 95%1.03–5.16]

Data is compared among APS patients with and without the defining phenotype

^a^*CL* cardiolipin, *β2GP1* beta2-glycoprotein1, *P-acid* phosphatidic acid, *PC* phosphatidylcholine, *PE* phosphatidylethanolamine, *PG* phosphatidylglycerol, *PI* phosphatidylinositol, *AN* annexin 5, *PS* phosphatidylserine, *PT* prothrombin

*Arterial thrombosis* was associated with accumulation of any 7 or more of the 20 aPLs evaluated in this study compared to the presence of 6 or less (odds ratio [OR] 4.1; confidence interval [CI] 95% 1.9–96, p = 0.001) and the sole presence of aPT IgG (OR 2.3 (CI 95% 1.1–5.1, p = 0.03). Patients diagnosed with primary APS were more prone to suffer from arterial thrombotic events compared to those with secondary APS (OR 2.2; CI 95% 1.1–4.5, p = 0.03).

*CNS manifestations* correlated with the co-presence of a specific profile of four aPLs: aPT, aPG, aPI and aAN of the IgG isotype (OR 2.6; CI 95% 1.1–6.3, p = 0.03).

*Venous thrombosis* was present among our well-defined APS cohort in 77/130 patients of which 72(93%) were sero-positive for at least one aPL tested herein. Notably, the presence of aPLs was highly associated with venous thrombosis in general, namely while comparing APS patients to our control groups, 72/77 patients with venous thrombosis were sero-positive for at least 1 aPL (either IgG or IgM) whereas only 7/40 healthy control or 16/58 healthy and sepsis control were positive (p < 0.001 for both comparisons respectively). Among our APS patients, venous thrombosis as a phenotype was not linked to any specific aPL, but rather an inverse correlation was found between this phenotype and aP-acid IgM (OR 0.3; CI 95% 0.1–0.9, p = 0.02).

*Latency from last thrombotic events* The time range from last thrombotic event in our cohort was 0.5 to 16 years with an average of 4.8 years. Event-free period (i.e. with no recurrence of thrombotic events) of more than 3 years was significantly associated with sero-positivity to either aPI IgG or aAN IgG (OR 3; CI 95% 1.08–8.1, p < 0.05 and OR 3.4; CI 95% 1.08–10.9, p < 0.05, respectively), as well as with a lower cumulative number of aPLs (7.6 ± 6 *vs.* 11 ± 6, p < 0.05) compared to patients that experienced recurrent thrombosis within this time.

*Pregnancy morbidity* was observed in 38/103 (37%) of female APS patients. A profile of 2 aPLs: aCL IgG and aPS IgG was linked with this phenotype (OR 2.9; CI 95% 1.3–6.5, p < 0.05) as well as the sole positivity of anti-AN IgG (OR 2.8; CI 95% 1.02–8, p = 0.05). Notably, APS-related pregnancy morbidity was also linked to arterial thrombosis (OR 3.3; CI 95% 1.5–7.2, p = 0.004), CNS morbidity (OR 3.9; CI 95% 1.7–9, p = 0.001), and secondary APS (OR 2.3; CI 95% 1.03–5.16, p = 0.04).

*Older age* allied with the presence of aP-acid IgM (51 y/o *or older*; p = 0.001) as well as with the profile of aβ2GP1 IgM and aCL IgM (46 y/o *or older;* p = 0.03) compared to sero-negative patients to these specific antibodies.

*Gender* was linked with specific aPLs: male with aCL IgG, aPG IgG, aPI IgG, aAN IgG and aPT IgG and female with aPE IgM (Table 4).

**Table 4:** Gender association with anti-phospholipids antibodies (aPLs) among patients with the anti-phospholipids syndrome (APS)

APLs associated with gender	Odds ratio (CI 95%) for female sex	Odds ratio (CI 95%) for male sex
aPE IgM	*2.5 (1.03–6)*	0.39 (0.16–0.96)
aCL IgG	0.3 (0.1–0.6)	*3.4 (1.6–7.4)*
aPG IgG	0.2 (0.1–0.5)	*2.2 (1–4.6)*
aPI IgG	0.2 (0.1–0.5)	*2.5 (1.1–5.4)*
aAN IgG	0.2 (0.1–0.4)	*3.0 (1.3–6.9)*
aPT IgG	0.1 (0.4–0.3)	*3.8 (1.7–8.5)*

*PE* phosphatidylethanolamine, *CL* cardiolipin, *PG* phosphatidylglycerol, *PI* phosphatidylinositol, *AN* annexin 5, *PT* prothrombin

*Concomitant autoimmune diseases* 67/130 (51.5%) of our APS cohort had a concomitant autoimmune disease, which in the vast majority was systemic lupus erythematosus, defining secondary APS. In comparison to primary APS, secondary disease correlated with the presence of aβ2GP1 IgG (OR 3.9; CI 95%:1.8–8.2, p = 0.04).

### Interactions between different aPLs

In the current study we observed interactions between most aPLs, as the presence of each aPL was statistically associated with sero-positivity of other aPLs of the same isotype (IgG or IgM; data not shown). An exception was noted for aPE of IgM isotype which was linked only with aβ2GP1 IgM (p < 0.05) and not with all other aPLs. Interestingly, the presence of LAC correlated with aPLs of the IgG subtype directed at CL, P-acid, PG, PI, PS, β2GP1, and PT (p < 0.05).

## Discussion

In this study we evaluated 20 “criteria” and “non-criteria” aPLs targeted at 10 different phospholipids or phospholipid-binding proteins in a cohort of well-defined “highly active serologically” APS patients (i.e. 84% were LAC positive and 55% “triple positive”) aiming to correlate these aPLs with clinical phenotypes of APS.

APS is a unique acquired thrombotic condition, which causes both arterial and venous thrombosis that may reoccur despite anti-coagulation therapy. Arterial and recurrent thrombosis are both considered to have a worse outcome, thus are usually followed by a more forceful therapy [9, 26]. Herein, we found that certain aPLs and/or aPLs profiles are associated with three APS thrombotic phenotypes: arterial thrombosis, CNS manifestations and recurrent thrombosis. Arterial thrombosis linked with the presence of any 7 or more aPLs compared to 6 or less. This stands in agreement with the notion that aPLs accumulation is an adverse marker of APS. The latter was put forward in 2007 by Bizzaro. et al. [27] and in 2011 by Pengo. et al. [28] defining “triple positivity” as a risk factor for thrombosis. Later on Otomo. et al. [9] evaluated criteria (aCL, aβ2GP1) and non-criteria (aPS/PT complex) aPLs documented aPLs accumulation as a prognostic factor, so did Cervera R. et al. [29] that demonstrated “non-criteria” aPLs (aPT, aPE, anti-vimentin etc.,) relation to disease severity.

Additionally, we found a tie between arterial thrombosis and the existence of aPT. The thrombotic-predictive value of aPT was formerly suggested in a 15 year longitudinal study of SLE patients [27] as well as in a prospective study conducted by Forastiero et al. [30]. Lately Zhang et al. [31] found, similar to our data, that aPT relates to arterial thrombosis. In contrast, a review of 11 studies including 1440 patients concluded that aPT assessment was non-contributory for routine APS laboratory workout [32]. These contradicting conclusions may result from different methods used for detection of aPT or different cohorts of patients assessed. But perhaps the most striking difference is the role looked at as for routine APS workout aPT appears to render no benefit while a plausible role for risk stratification and phenotyping of “well-defined” APS and SLE patients may be suggested.

APS commonly affects the central nervous system (CNS) manifesting as stroke, seizures, dementia, cognitive dysfunction, chorea, migraine, psychosis, demyelination etc. [4, 33]. In this study, for the first time to the best of our knowledge, an association between APS-related CNS manifestations and a specific profile of four aPLs namely the co-presence of aPT aPG, aPI and aAN of the IgG isotype was observed, of which, like aPT also aAN and aPI were interrelated with thrombosis [34]. Annexins are a group of 12 regulatory proteins that are involved in vesicle trafficking, calcium signaling, cell growth, division, and apoptosis. Annexin 2 and 5 have an affinity to phospholipids, and antibodies directed at these proteins were found in patients with either arterial or venous thrombosis. Likewise, anti-phosphatidylinositol antibodies were significantly associated with thrombosis among APS and SLE patients [35].

One of the most difficult phenotypes of APS is the ‘recurrent thrombosis’ one. Approximately 20% of APS patients will experience recurrence within 3.4 to 16.3 years, depending on their treatments with antiplatelet, anticoagulant, or a combination of these therapies [36]. Currently, there are no established risk factors for ‘the thrombotic recurrence phenotype’ though the plausible role of aPLs has been suggested, and may eventually allude to enhanced therapy for patients at risk [37–39]. Herein, we found that a low recurrence rate, defined as thrombosis free period of more than 3 years, links with a lower accumulative number of aPLs, further supporting the perception that more aPLs allied with a worst outcome.

APS is the most frequently acquired risk factor for recurrent pregnancy losses, ischemic placental dysfunction, fetal growth restriction, preeclampsia, premature birth and intrauterine death [14, 40]. Apart from the thrombotic variants of APS, obstetric APS (OAPS) is probably the most common phenotype. Obstetric morbidity may be the only presentation of APS or may co-exist with thrombotic and non-thrombotic phenotypes [41]. In the current study, we found that obstetric morbidity was linked with CNS and arterial thrombotic phenotypes, which stands in agreement with other reports such as a recent study by Gris et al. [42] documenting OAPS association with mental disorders. Besides, we found OAPS to be linked with a profile of two aPLs: aCL and aPS or the sole presence of aAN of the IgG isotype. Recently, aCL was found to be the most common aPL present in a large cohort of 750 pregnancies [43]. Equally, aPS was linked with OAPS in several studies and in particular, monoclonal aPS antibodies were found to reduce yolk sac growth in animal models as well as placental trophoblastic cell growth and proliferation in humans [44]. In contrast to aCL and aPS, the role of aAN in OAPS is yet controversial. The latter was linked with recurrent pregnancy morbidity and losses in some studies [35, 45] but this association was not ascertained in others [41, 46, 47]. Still, the prediction of aPL related pregnancy morbidity is an issue of great debate, especially among women defined as “only aPLs carriers” or those with less than three early miscarriages. Thus, although our results require further studies, the idea that certain aPLs or aPLs profile may be used as a marker of pregnancy morbidity is intriguing.

There is a strong link between aPLs and venous thrombosis as was described in numerous studies as well as the current one while comparing APS patients to healthy subjects and patients diagnosed with sepsis. However, none of the aPLs studied herein was specifically linked with venous thrombosis among APS patients. In other words the vast majority of patients with venous thrombosis were aPL sero-positive and no differences were documented compared to APS patients that did no exhibit venous thrombosis. In contrast, a striking observation in this study was the inverse association of anti P-acid antibody of the IgM isotype with venous thrombosis. The anti P-acid antibody was rarely studied as a single antibody and for the best of our knowledge this is the first report of such inverse association. Of note anti P-acid was linked with thrombosis in several studies which evaluated mostly IgG antibodies and regarded thrombosis in general both arterial and venous. In a recent study of sero-negative APS patients, anti P-acid was linked with fetal losses and not with thrombosis [48]. Further studies are required to verify such an inverse correlation. Interestingly in this study the anti-P-acid IgM, as well as other IgM antibodies were linked with older age.

Last but not least, from the diagnostic perspective, the LIA was easy, efficient and with good sensitivity and specificity while employing cutoffs ascertained by healthy controls. This method enabled the discrimination of aPL found in APS patients from those in asymptomatic carriers as reported previously [24]. Within our controls, some aPLs were numerically more prevalent among septic control patients compared to healthy subjects, but a significant difference was observed only for anti-β2GP1 IgG. The latter transient appearance during infection was formerly reported [49, 50]. Furthermore, we observed interactions between different aPLs of the same isotype. Similar observations have been reported and led to the hypothesis that broad aPLs positivity may be due to epitope spreading [51].

Our study has several limitations, as our cohort included “serologically active” APS patients (84% were LAC positive and 55% triple positive) that fulfill the criteria for APS, which could cast doubt on the implication of our results to patients with “lower serological activity” (e.g. single low titer aPL). A selection bias was inevitable as all patients had a least one clinical manifestation of APS to fulfill its classification criteria. Noteworthy, this study aimed to define subtypes of APS rather than use aPL for diagnosis or classification of disease. Additionally, aPLs were evaluated in this study using a single blood sample from each subject and a single method of detection. The former do not allow estimations of aPL profiles at the time of events, but rather a retrospective clinical correlation, as well as the lack of long term follow up, nor the role of other aPLs or aPL-complexes as anti-PS/PT (only PS and PT separately) or anti-domain-1 of β 2GPI. The latter two aPL may add value to the assessment of APS patients requiring further studies. Using multiple comparative methods for detection of aPLs is of significance, hence the use of the line dot blot (LIA) semi quantitative method as a single method is a limitation. However, all patients included in this study were originally criteria aPL positive and recently Thaler et al. demonstrated similar results for detection of non-criteria aPL by the LIA method compared to ELISA [52]. Thus, we assume that there will be no significant differences by ELISA to our results by LIA. Moreover, Thaler et al [52] reported a higher sensitivity of the LIA technique for aPL recognizing anionic phospholipids. Lastly, the aim of our study was to evaluate APS profiles, therefore correlation with a single criteria or non-criteria manifestation (e.g. intra uterine fetal death of thrombocytopenia) was not evaluated.

## Conclusions

Herein, we report that criteria and non-criteria aPLs and/or aPLs profiles are statistically linked with APS phenotypes such as arterial thrombosis, CNS manifestations, recurrent thrombosis and obstetric APS. Additionally, we found that accumulation of these aPLs is associated with more severe variants of diseases namely arterial and recurrent thrombosis. Our data suggest that evaluating a broad spectrum of aPLs may enable defining APS phenotypes and, thus, may have a future role in precision choice of therapy for this autoimmune multifaceted disease.

## Data Availability

The clinical, serological data used to support the findings of this study are included within the article; further findings are available from the corresponding author upon request.
